# Acute Alcohol Consumption and Secondary Psychopathic Traits Increase Ratings of the Attractiveness and Health of Ethnic Ingroup Faces but Not Outgroup Faces

**DOI:** 10.3389/fpsyt.2015.00025

**Published:** 2015-02-19

**Authors:** Ian J. Mitchell, Steven M. Gillespie, Monica Leverton, Victoria Llewellyn, Emily Neale, Isobel Stevenson

**Affiliations:** ^1^School of Psychology, University of Birmingham, Birmingham, UK

**Keywords:** alcohol, psychopathy, attractiveness, health, ingroup, outgroup, prepotent, response inhibition

## Abstract

Studies have consistently shown that both consumption of acute amounts of alcohol and elevated antisocial psychopathic traits are associated with an impaired ability for prepotent response inhibition. This may manifest as a reduced ability to inhibit prepotent race biased responses. Here, we tested the effects of acute alcohol consumption, and elevated antisocial psychopathic traits, on judgments of the attractiveness and health of ethnic ingroup and outgroup faces. In the first study, we show that following acute alcohol consumption, at a dose that is sufficient to result in impaired performance on tests of executive function, Caucasian participants judged White faces to be more attractive and healthier compared to when sober. However, this effect did not extend to Black faces. A similar effect was found in a second study involving sober Caucasian participants where secondary psychopathic traits were related to an intergroup bias in the ratings of attractiveness for White versus Black faces. These results are discussed in terms of a model which postulates that poor prefrontal functioning leads to increases in ingroup liking as a result of impaired abilities for prepotent response inhibition.

## Introduction

The term psychopathy refers to a severe disorder of personality that is characterized by a callous disregard for others, a cunning and manipulative interpersonal style, and a lack of remorse or guilt (1, 2). In addition to these affective and interpersonal features, antisocial behavior has also been recognized as a core component of the psychopathy construct (3). Although psychopathic features are typically assessed among offending samples using the psychopathy checklist-revised (PCL-R) (1, 2), traits of the disorder can nonetheless be examined among sub-clinical populations using self-report personality inventories. These scales typically assess the affective/interpersonal features, including selfishness and a lack of care for others, and the lifestyle/antisocial features of the disorder, including recklessness and impulsivity. These traits can be assessed using the Levenson self report psychopathy scale (LSRP) (4), and are referred to as “primary” and “secondary” psychopathic traits, respectively.

It is argued that while the callous-unemotional (CU) traits of psychopathy have a strong genetic basis, the lifestyle/antisocial features have a much stronger basis in the environment. For example, Viding et al. (5) found a substantial genetic influence for antisocial behavior among twin pairs at age 7, but only in the presence of high CU traits. Similar results have also been observed at age 9 (6) and indicate that antisocial behavior in the absence of CU traits is better explained by non-shared environmental influences. These findings are consistent with claims that traumatic early environments can result in later antisocial behavior in the absence of CU traits. For example, it has been shown that developmental trauma can result in perturbed development of several brain areas, including territories of the prefrontal cortex (PFC) (7–9). Abnormalities of prefrontal structure and function have also been identified in individuals with antisocial behavior (10–12), and the PFC plays an important role in empathy (13, 14), morality (15), and prepotent response/impulse inhibition (16–18). These findings therefore support a link between PFC structure and function and antisocial behavior.

Interestingly, many of the social-cognitive impairments associated with antisocial behavior can also be observed following acute alcohol consumption. For example, alcohol consumption is associated with transient decreases in moral maturity (19), and reduced empathic responses toward a female rape victim (20). Furthermore, intoxicated participants also show problematic prepotent response inhibition (21–23), and reduced frontal event-related potentials (ERPs) (24). These impairments are presumed to reflect the fact that acute alcohol consumption exerts a profound, but reversible, effect on the functioning of the PFC (25), and compromises executive function (26–28).

Impaired inhibition of prepotent responses following alcohol have also been revealed in terms of race biased responding. For example, Bartholow et al. (29) showed that depleted self-regulatory processes while intoxicated were associated with a reduced ability to inhibit racial bias. Similarly, Schlauch and colleagues found that participants made more race biased responses under speeded conditions following alcohol consumption compared to when sober (30). These findings suggest that reduced PFC function can lead to impaired inhibition of race biased responding. Similar findings have also been reported with sober participants during the inhibition of race biased responses under conditions of increased demands on executive processes (31, 32), and during brain imaging studies (33). Moreover, findings from developmental studies have shown that ingroup favoritism can be observed among preschool children who are yet to fully develop the higher level, executive abilities required to inhibit race biased responses (34), while out-group derogation only appears later, typically after the age of 6 (35). As such, we would suggest that ingroup liking may reflect a prepotent response.

Based on the finding of race biased responding following acute alcohol consumption, three hypotheses may be generated (1) that alcohol consumption is related to an increase in ingroup liking, (2) that alcohol consumption is related to an increase in outgroup derogation, or that (3) alcohol consumption leads to increases in both ingroup liking and outgroup derogation. These hypotheses can be tested using simple experimental approaches. For example, it has been shown that ratings of attractiveness are sensitive to the consumption of alcohol, and there is evidence to suggest that acute alcohol consumption can result in enhanced judgments of attractiveness, especially when judgments are made of the opposite sex (36–38). However, the effects of ethnicity on these findings remain unknown. Alcohol may also similarly affect judgments that are related to attractiveness, including perceived healthiness. For example, a series of experimental studies have established that there is a close relationship between our subjective judgments of others attractiveness and our predictions of how healthy those individuals are (39–41). Furthermore, this link has been shown to hold when considering an individual’s actual longevity (42). Thus, it can be predicted that impaired functioning of the PFC, as observed following acute alcohol consumption, or in relation to antisocial psychopathic traits, may lead to more positive ratings of attractiveness and health for ethnic ingroup members (ingroup liking), or alternatively, reduced ratings of the attractiveness and health of ethnic outgroup members (outgroup derogation), or both of these.

In this paper, we tested these hypotheses across two separate studies. In Study 1, we investigated the effects of acute alcohol consumption on ratings of healthiness and attractiveness of ethnic ingroup and outgroup faces in a sample of white Caucasian participants. We hypothesized that participant’s ratings of the attractiveness and health of ethnic ingroup, but not outgroup members, would be more positive while intoxicated. In Study 2, we examined the hypothesis that individuals scoring highly on a trait measure of secondary psychopathic traits would make more positive ratings of the attractiveness and health of ethnic ingroup relative to outgroup faces.

## Study 1: Acute Alcohol Consumption and Judgments of Attractiveness and Health of Ethnic In- and Out-Group Faces

### Methods

#### Participants

Twenty-six participants (17 female, 9 male) ranging in age from 19 to 22 years (*M* = 20.72, SD = 0.89) took part in the study. All were British, Caucasian, undergraduate students at a British university. All participants gave informed consent while sober. Ethical permission for the study was granted by the University of Birmingham Science, Technology, Engineering, and Mathematics Ethical Review Committee.

#### Materials and tests

Two tests of executive function were used: the color Stroop, and an abridged FAS word fluency test (43). The former is sensitive to inhibitory deficits (44) while the latter can detect alcohol induced deficits in executive function (27). The color Stroop consisted of a white A4 page showing 80 color naming words printed in different colored ink. Participants were required to say the ink color that color name words were printed in, as quickly and as accurately as possible. For the word fluency test, participants were asked to say as many words as they could think of beginning with the letter F in 60 s.

Facial stimuli for ratings of attractiveness and health consisted of eight colored photographs presented together on a single page. Each photograph was a headshot of a full face displaying a neutral expression, set against a white background. The faces were either White/Black, male/female, and unfamiliar/celebrity. Each of the possible combinations of face characteristics was represented once. The photographs were selected such that each pair of White/Black faces was approximately matched in terms of attractiveness. The celebrities were typically considered to be far more attractive than the unknown individuals, irrespective of ethnicity or sex. The unknown faces were taken from the NimStim face database (45) (http://www.macbrain.org/resources.htm). The pictures of celebrities were taken from a variety of websites.

#### Procedure

Both the alcohol and no-alcohol conditions were conducted at drinks parties held within student accommodation. Testing in the alcohol condition began once the participant had consumed four units of alcohol and after a 1 h period from joining the social event. The alcohol consumption parameters were kept relatively constant with respect to dose and time of testing, although the levels of blood alcohol were not carefully monitored. This in part reflected the desire to maintain a more naturalistic setting for the experiment. In the sober condition, participants had to have abstained from drinking alcohol for the 24 h period preceding the event and during testing. The order of conditions was randomized and the interval between conditions was 7–9 weeks. The tests of executive function were administered prior to the attractiveness and health ratings of the faces being completed. Participants were then shown the face stimuli and asked to rate them in terms of attractiveness and perceived healthiness using an 11-point Likert scale (0 = *very unattractive/unhealthy*, 10 = *very attractive/healthy*).

### Results

#### Data analytic strategy

Paired samples *t*-tests were used to compare differences in the number of errors and response times on the color Stroop, and differences in the number of words generated on the FAS word fluency test, while sober, and while drunk. To examine the acute effects of alcohol consumption on ratings of attractiveness and health, for White and Black face stimuli, we used two repeated measures ANOVAs with sobriety (sober, intoxicated), race (White, Black), and familiarity (familiar, unfamiliar) as the factors.

#### Tests of executive functioning

Performance on the Stroop color word test was poorer following alcohol, with participants making more errors and taking 14.4% longer to complete the task. Performance on the abridged version of the FAS word fluency test was also poorer following alcohol with participants generating 22% fewer words in the alcohol condition relative to the sober condition. Paired *t*-tests showed each of these differences to be highly significant, implying that the dose of alcohol was sufficient to impair prefrontal functioning (see Table 1).

**Table 1:** **Effects of acute alcohol consumption on participants’ (*N* = 25) executive function**.

	Sober (*M* ± SE)	Alcohol (*M* ± SE)	*t*-Test (*t, p)*
**Color stroop**
Errors	0.48 ± 0.165	3.8 ± 0.735	−4.61, <0.001
Time (min)	1.04 ± 0.047	1.19 ± 0.038	−3.94, <0.001
**FAS word fluency**
Words generated	21.68 ± 0.770	16.92 ± 0.975	4.77, <0.001

#### Effects of alcohol intake on attractiveness ratings

**Figure 1:**
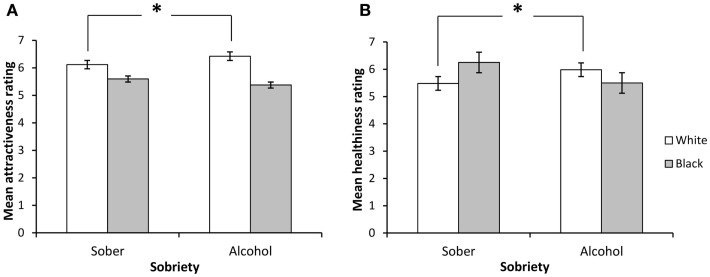
**Effects of sobriety state on ratings of (A) attractiveness and (B) healthiness of ethnic ingroup and outgroup faces**. Consumption of acute amounts of alcohol was associated with increases in ratings of attractiveness (*p* < 0.05) and healthiness (*p* < 0.05) of ethnic ingroup members, but not outgroup members, compared to while sober.

Figure 1 shows attractiveness and healthiness ratings for White and Black faces while sober and following alcohol. In spite of the stimuli being selected to ensure that the faces from the two ethnicities were approximately matched in terms of attractiveness, a main effect of race nonetheless showed that White face stimuli (*M* = 6.27, SD = 0.61) were rated as significantly more attractive than Black face stimuli (*M* = 5.49, SD = 0.99) *F*(1, 25) = 18.96, *p* < 0.001, *p*η^2^ = 0.43. As expected, a main effect of familiarity showed that, across both sobriety and race, celebrity faces were rated as significantly more attractive (*M* = 7.29, SD = 0.95) than unfamiliar faces (*M* = 4.47, SD = 0.84) *F*(1, 25) = 148.95, *p* < 0.001, *p*η^2^ = 0.86. Crucially, we also showed a significant interaction of sobriety and race *F*(1, 25) = 7.98, *p* < 0.01, *p*η^2^ = 0.24. In order to better understand this interaction, we used paired sample *t*-tests to examine attractiveness ratings for White and Black faces while sober and following alcohol. Results showed that participants rated White faces as more attractive after drinking alcohol compared to when sober (*t* = 2.17, *p* < 0.05) (see Figure 1). The effect of alcohol intake on attractiveness ratings for Black face stimuli failed to reach significance (*t* = −1.27, *p* > 0.05).

#### Effects of alcohol intake on health ratings

As expected, the analysis of health ratings for White and Black faces showed that celebrity faces (*M* = 7.10, SD = 1.02) were rated as more healthy than unfamiliar faces (*M* = 4.50, SD = 1.12) across both sobriety and race *F*(1, 25) = 123.16, *p* < 0.001, *p*η^2^ = 0.83. However, we observed no significant main effect of race *F*(1, 25) = 1.08, *p* = 0.48, *p*η^2^ = 0.02, with similar health ratings for White (*M* = 5.73, SD = 0.67) and Black (*M* = 5.88, SD = 1.29) faces. Critically, the analysis revealed a significant interaction of sobriety state and race *F*(1, 27) = 15.30, *p* < 0.01, *p*η^2^ = 0.38. Paired samples *t*-tests showed that this interaction was driven by a tendency to rate White faces as more healthy following alcohol compared to when sober (*t* = −2.17, *p* < 0.05) (see Figure 1). Health ratings for Black faces on the other hand were found to be similar while sober and while drunk (*t* = 1.27, *p* > 0.05).

#### Relationship between ratings of attractiveness and health

We used Pearson’s correlation coefficient to examine the relationship between ratings of attractiveness and health for White and Black faces while sober and while drunk. Analyses showed that across all conditions, there were significant positive correlations between attractiveness and health (all *r* > 0.48, *p* < 0.05).

### Discussion

Study 1 aimed to investigate the hypothesis that acute alcohol consumption would influence Caucasian participants’ judgments of attractiveness and health for White and Black face stimuli. It was predicted that participants’ judgments of attractiveness and healthiness for White face stimuli would be more positive while intoxicated compared to while sober. In contrast, we predicted that there would be either no change, or that participants’ judgments of Black faces would be more negative following alcohol.

The principal finding from Study 1 was that the acute consumption of modest amounts of alcohol by Caucasian participants was associated with increased ratings of the attractiveness and health of White face stimuli. Ratings of Black face stimuli, however, remained unchanged following alcohol consumption. These findings are consistent with other findings which show that alcohol consumption is associated with increases in ratings of attractiveness (36–38), although our findings suggest that this effect is specific to faces of ethnic ingroup members. These findings provide support for the hypothesis that alcohol consumption leads to an increase in the intergroup bias. Furthermore, our findings suggest that this effect may be driven by increases in ingroup liking. We found no support for the hypothesis that consuming modest amounts of alcohol would be associated with increased outgroup derogation.

The findings from Study 1 provide evidence that judgments of attractiveness and health of face stimuli may be altered following acute alcohol consumption. The finding of reduced performance on the Stroop color word task and the FAS word fluency test, while intoxicated compared to while sober, confirmed that participants showed impaired functioning of the PFC following the consumption of acute amounts of alcohol. We would suggest that the findings of Study 1 may therefore reflect the effects of acute alcohol consumption on the functioning of the PFC. Given the similarity in the social-cognitive impairments observed following the consumption of modest amounts of alcohol, and those observed in relation to antisocial behavior, it may be hypothesized that similar results would be found in relation to a measure of antisocial personality traits. Thus, in Study 2, we aimed to examine the relationship of secondary psychopathic traits, characterized by recklessness and impulsivity, with judgments of attractiveness and health for White and Black face stimuli.

Although secondary psychopathic traits are associated with impairments of the PFC, the affective/interpersonal features of the psychopathic personality, also termed “primary” psychopathic traits by Levenson et al. (4), are linked with hypoactivity of the amygdala (46). We therefore controlled for the effects of primary psychopathic traits to investigate the unique influence of secondary psychopathic traits on judgments of attractiveness and health. We hypothesized that impaired functioning of the PFC associated with secondary psychopathic traits would be reflected in a more prepotent response style. Based on the findings of Study 1, and findings from developmental studies which suggest that ingroup liking reflects a developmentally earlier response style, we predicted that secondary psychopathic traits would be associated with more positive judgments of the healthiness and attractiveness of White face stimuli, but not Black face stimuli.

## Secondary Psychopathic Traits and Judgments of the Attractiveness and Health of Ethnic In- and Out-Group Faces

### Methods

#### Participants

Thirty participants (26 female, 4 male), ranging in age from 18 to 35 years (*M* = 19.67, SD = 3.06) took part in the study. All were British, Caucasian, undergraduate students at a British university. All participants gave informed consent. The study was approved by the University Ethical Review Committee.

#### Materials and tests

The set of facial stimuli used in Study 1 was also used here. Psychopathic personality traits were measured using the LSRP (4). The LSRP was designed for the measurement of psychopathic personality traits in non-institutionalized populations. The scale consists of a 16 item primary psychopathy subscale, and a 10 item secondary psychopathy subscale. While the primary subscale taps the selfish and uncaring characteristics associated with Factor 1 of the PCL-R (1, 2), the secondary subscale measures the behavioral and lifestyle features associated with Factor 2, including impulsivity and poor behavioral control. Participants respond to each item on a 4-point Likert scale, with responses ranging from “*disagree strongly*” to “*agree strongly*.” Adequate internal validity of the LSRP has been demonstrated in a large sample of non-offenders, with a Cronbach’s alpha of 0.82 for the primary subscale, and 0.63 for the secondary subscale, which was considered adequate for a 10 item scale (4).

#### Procedure

Testing took place in a laboratory at UK university. Participants were first shown the face stimuli and asked to rate them in terms of attractiveness and perceived healthiness using an 11-point Likert scale (0 = *very unattractive/unhealthy*, 10 = *very attractive/healthy*). After completing ratings of face stimuli participants were asked to complete the LSRP.

### Results

#### Data analytic strategy

We used two repeated measures ANCOVAs with the factors race (White, Black) and familiarity (unknown, celebrity), with primary and secondary psychopathic traits included as covariates, to examine the effects of these traits on ratings of attractiveness and health for face stimuli.

#### Effects of psychopathic traits on attractiveness ratings

**Figure 2:**
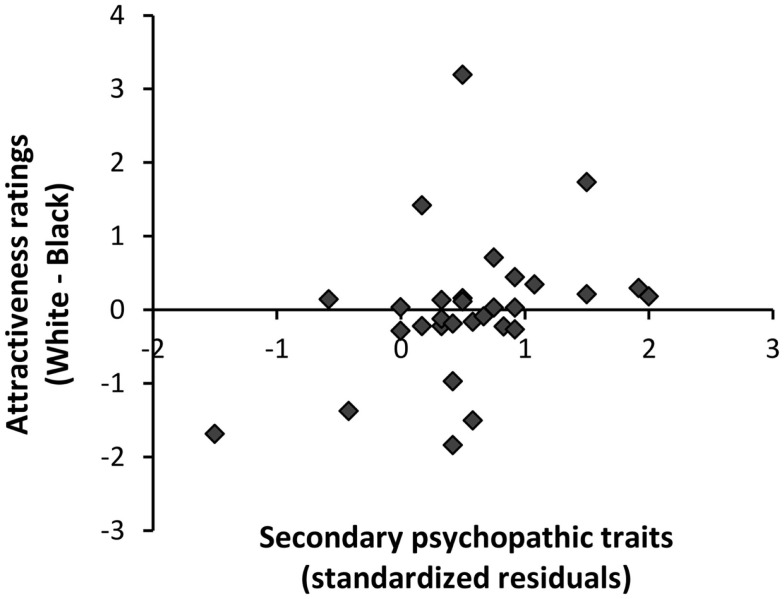
**Relationship of secondary psychopathic traits with judgments of attractiveness for ethnic ingroup relative to ethnic outgroup faces**. A partial correlation, controlling for primary psychopathic traits, showed that increasing secondary psychopathic traits were associated with more positive ratings of attractiveness for ethnic ingroup faces relative to ethnic outgroup faces (*r* = 0.38, *p* < 0.05).

As expected, the analysis revealed a significant effect of familiarity, with celebrity (*M* = 7.11, SD = 1.02) faces rated as more attractive than unfamiliar (*M* = 4.91, SD = 1.07) faces *F*(1, 27) = 40.40, *p* < 0.001, *p*η^2^ = 0.60. The main effect of race however was non-significant *F*(1, 27) = 3.23, *p* = 0.08, *p*η^2^ = 0.11, with similar attractiveness ratings observed for White (*M* = 6.28, SD = 0.97) and Black faces (*M* = 5.73, SD = 0.92). Critically, we revealed an interaction of race with secondary psychopathic traits *F*(1, 27) = 4.45, *p* < 0.05, *p*η^2^ = 0.14. To better understand this interaction, we computed the difference in attractiveness for White and Black faces (attractiveness ratings for White faces–Black faces) collapsed across familiarity. A partial correlation of secondary psychopathic traits, controlling for primary psychopathic traits, with attractiveness ratings for White relative to Black faces showed that secondary psychopathic traits were associated with elevated ratings of attractiveness for White compared to Black faces (*r* = 0.38, *p* < 0.05) (see Figure 2).

#### Effects of psychopathic traits on health ratings

We showed that there was no significant main effect of race on health ratings *F*(1, 27) = 0.00, *p* = 0.99, *p*η^2^ = 0.00, with similar ratings of healthiness for both White (*M* = 6.59, SD = 0.81) and Black (*M* = 6.36, SD = 0.92) faces. However, there was a significant main effect of familiarity *F*(1, 27) = 27.16, *p* < 0.001, *p*η^2^ = 0.50, with celebrity faces (*M* = 7.57, SD = 0.92) rated as more healthy than unfamiliar faces (*M* = 5.39, SD = 1.05). We observed no significant effects of secondary psychopathic traits on health ratings for White and Black faces *F*(1, 27) = 1.80, *p* > 0.05, *p*η^2^ = 0.06.

#### Relationship between ratings of attractiveness and health

Consistent with the results of Study 1, we showed that health and attractiveness ratings were highly positively correlated across all stimulus categories (all *r* > 0.51, *p* < 0.01), suggesting that increases in perceived healthiness of skin were associated with more positive judgments of facial attractiveness.

### Discussion

Study 2 aimed to investigate the effects of psychopathic personality traits on judgments of attractiveness and health for White and Black ethnicity face stimuli. It was hypothesized that the problematic prepotent response inhibition associated with secondary psychopathic traits would be associated with a bias in health and attractiveness judgments for White and Black ethnicity faces. We predicted that heightened levels of secondary psychopathic traits would be associated with more positive judgments of the health and attractiveness of White relative to Black face stimuli.

Results showed that more elevated levels of secondary psychopathic traits were associated with a tendency toward more positive judgments of attractiveness for White relative to Black face stimuli. These findings are consistent with those observed in Study 1 and suggest that problematic prepotent response inhibition may be associated with an increase in ingroup liking. However, although we showed that healthiness and attractiveness judgments were positively correlated, there was no evidence that secondary psychopathic traits effected healthiness judgments for White or Black ethnicity face stimuli.

## General Discussion

In this paper, we report the results of two separate studies that aimed to examine the effects of potentially impaired prefrontal functioning on judgments of healthiness and attractiveness for White and Black ethnicity face stimuli. In Study 1, we asked participants to provide attractiveness and health judgments for White and Black faces under two conditions, while sober and following acute alcohol consumption. Results showed that following acute alcohol consumption, participants provided more positive judgments of attractiveness and health for White, but not Black face stimuli. Findings from Study 2 showed that elevated secondary psychopathic traits, which are characterized by recklessness and impulsivity, were linked with more positive judgments of attractiveness for White relative to Black face stimuli.

The effects of alcohol on judgments of attractiveness and health may reflect the potent effects of alcohol on the functioning of the PFC (26, 27). In Study 1, although levels of blood alcohol were not carefully monitored, we confirmed that consumption of alcohol did elicit significant changes in executive function. The participants generated 22% fewer words on the FAS word fluency task, and were 14% slower and made eight times more errors on the Stroop color word task in the alcohol condition relative to when sober. Thus, the experimental manipulation was clearly successful in meeting the objective of compromising prefrontal functioning. Furthermore, the poorer performance on the Stroop task implies that alcohol consumption had reduced the ability to inhibit prepotent responses.

The current finding that alcohol increased healthiness and attractiveness judgments for White face stimuli is consistent with previous reports of enhanced attractiveness judgments, especially for the opposite sex, following alcohol consumption (36–38). However, the ethnicity of the facial stimuli used was not systematically manipulated in these earlier studies. The mechanism underlying these alcohol induced shifts in judgments of attractiveness are not completely understood but may involve an impaired ability to judge facial symmetry (37), a facial feature that is known to be a strong determinant of attractiveness (47, 48). This impaired ability could lead to faces appearing more symmetrical following alcohol compared to when sober, and this in turn could affect judgments of attractiveness. However, an impaired ability to judge facial symmetry may be expected to have an equal effect on judgments of White and Black face stimuli, but this was not the case.

An alternative mechanism that may account for our findings in Study 1 relates to the release from inhibition of a prepotent response. It has consistently been shown that alcohol exerts a profound, but reversible, effect on the functioning of the PFC (25), compromising executive function (26–28), and impairing prepotent response inhibition (29, 30). An impaired ability for prepotent response inhibition also has implications for race biased responding, with executive processes and areas of the PFC shown to play a critical role in inhibiting race biased responses (33). The finding of more positive ratings of healthiness and attractiveness following alcohol consumption may therefore reflect the unmasking of a prepotent response toward ingroup liking. This finding is consistent with the results of developmental studies which show that ingroup liking can be found among children at six years of age (34), before the PFC and executive abilities required for response inhibition have fully developed. In contrast, a pattern of outgroup derogation has been found among older children (35), suggesting that dislike of outgroups presents at later stages of development. Impaired prepotent response inhibition following alcohol consumption may therefore mirror the finding of ingroup liking among young children.

These findings from Study 1 were also supported by the findings of Study 2, which showed an intergroup bias in ratings of attractiveness for White and Black ethnicity faces. Similar to Study 1, we showed that secondary psychopathic traits were associated with elevated judgments of attractiveness for White relative to Black faces. These findings may reflect a tendency to view members of the ingroup more positively relative to members of outgroup. Although such automatic judgments may normally be under the influence of prefrontal cortical mechanisms, these inhibitory effects may be impaired among individuals with elevated secondary psychopathic traits. In support of this finding, we have previously revealed an exaggerated intergroup bias among individuals who score highly for these traits across a series of economical decision making games (49). In this work, we showed that high relative to low scoring secondary psychopathic traits participants offered more generous monetary amounts to members of an ingroup, relative to an outgroup, as identified through affiliation to UK universities. Although the extent to which these findings may be extended to other aspects of social cognition during intergroup interactions remains unclear, the results of Study 2 suggest that the findings of Gillespie et al. are not restricted to economical decision making games. Furthermore, the results of Study 2 presented here, together with the findings of Gillespie et al. (49), demonstrate an intergroup bias in relation to secondary psychopathic traits during interactions with members of the same or other university, and also while rating members of an ethnic ingroup or an ethnic outgroup.

It should be noted that the current study was limited to testing just Caucasian participants and the facial stimuli were restricted to White and Black faces. Whether this effect extends to facial stimuli of other ethnicities remains to be determined. Similarly, it is not known whether Black individuals would respond in an equivalent manner and show increased health and attractiveness ratings for images of Black ingroup faces relative to White outgroup faces. However, it has been shown by Hart et al. (50), who monitored amygdala activation following the presentations of White and Black facial stimuli, that both Caucasian and Black participants showed equivalent neural activity in response to ethnic ingroup, but not outgroup faces.

Also, the observed effect of secondary psychopathic traits may be tempered by potentially low levels of these traits in non-offending populations. For example, Coid et al. noted a low prevalence of psychopathy in the general household population of Great Britain (51), and there may also be differences in the distribution (51) or manifestation (2) of psychopathic traits between males and females. Thus, generalizations from a mixed sex, undergraduate sample to clinical forms of psychopathy should be made with considerable caution. Other difficulties may reflect potential pitfalls of the self-report measurement of psychopathic personality. Lilienfeld and Fowler (52) note that a deceitful and manipulative interpersonal style is recognized as a hallmark feature of the psychopathic personality, and that this may pose problems during self-report of psychopathic traits. However, based on the results of a meta-analysis, Ray et al. concluded that self-report psychopathy scales provide a valid indication of psychopathic personality traits (53).

The current findings may give some insights into the effects of alcohol and personality traits on one’s social behavior. Both acute consumption of alcohol and personality traits associated with poor impulse inhibition may contribute toward feelings of ingroup liking. These effects may be apparent in our ratings of how attractive and healthy other individuals are, and may therefore have consequences for conviviality and mate choice. While Study 1 showed increased healthiness and attractiveness ratings for members of the ingroup following alcohol consumption, in Study 2, we showed that secondary psychopathic personality traits are linked with more positive attractiveness judgments for the in- relative to the outgroup. Although the ways in which alcohol consumption and secondary psychopathic traits affect other areas of social cognition during intergroup social interactions remain unclear, these results suggest that factors associated with impaired prepotent response inhibition may contribute to social divides and race biased responding during intergroup interactions. These results also suggest that studying the effects of controlled alcohol intake may be of use in understanding the effects of impaired functioning of the PFC on social cognition and behavior. Furthermore, studying the effects of acute alcohol consumption may inform the development of biological models for understanding prepotent response inhibition in relation to antisocial behavior.

## Conflict of Interest Statement

The authors declare that the research was conducted in the absence of any commercial or financial relationships that could be construed as a potential conflict of interest.
